# The prognostic value of prognostic nutritional index in advanced cancer receiving PD‐1/L1 inhibitors: A meta‐analysis

**DOI:** 10.1002/cam4.4668

**Published:** 2022-03-16

**Authors:** Pengfei Li, Yutian Lai, Long Tian, Qinghua Zhou

**Affiliations:** ^1^ Department of Lung Cancer Center West China Hospital, Sichuan University Chengdu China

**Keywords:** malignancy, PD‐1/L1 inhibitors, prognosis, prognostic nutritional index

## Abstract

**Purpose:**

To study the prognostic value of the prognostic nutritional index (PNI) in advanced cancers receiving programmed death‐1/programmed death‐ligand 1 (PD‐1/L1) inhibitors.

**Methods:**

Online electronic databases were comprehensively searched and available literature was retrieved. We extracted available data from included researches and pooled the hazard ratios (HRs) and 95% confidence intervals (CIs) to learn the prognostic value of PNI on overall survival (OS) or progression‐free survival (PFS); and meanwhile calculated the relative risk (RR) with 95% CI to study the relationship between PNI and treatment efficacy (objective response rate [ORR] or disease control rate [DCR]) in late staged cancer receiving PD‐1/L1 inhibitors.

**Results:**

Nine studies were finally selected for this meta‐analysis. We obtained data regarding PNI on OS from all nine studies, and the pooled HR was 2.31 (95% CI 1.81–2.94, *p* = 0.000), showing a correlation between low PNI and worse OS. Eight studies reported a relationship between PNI and PFS, and combined results revealed shorter PFS in patients with lower PNI, with an HR of 1.75 (95% CI 1.40–2.18, *p* = 0.000). Four studies explored the association between PNI and ORR and two studies explored the influence of PNI on DCR. An association between PNI and ORR (RR = 0.47, *p* = 0.003) was observed, while no association between PNI and DCR (RR = 0.49, *p* = 0.103) was observed by pooling these studies.

**Conclusion:**

In summary, this meta‐analysis indicated that a lower PNI was significantly correlated with decreased OS and PFS and played adverse roles in ORR in advanced cancer patients receiving PD‐1/L1 inhibitors. Therefore, PNI could be promising for predicting prognosis and treatment response in advanced malignancies treated with PD‐1/L1 inhibitors.

## Graphical abstract

This meta‐analysis accessed the prognostic value of PNI on survival on advanced or inoperable cancers treated with PD‐1/L1 inhibitor. The results of this meta‐analysis revealed that low PNI was significantly correlated with poorer overall survival and progression free survival of advanced cancers treated with PD‐1/L1 inhibitor.

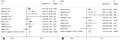

## 1 INTRODUCTION

Recent advances in programmed death‐1/programmed death‐ligand 1 (PD‐1/L1) inhibitors therapy have substantially revolutionized the cancer therapeutic status by improving the survival of certain advanced or metastatic cancers.^1^, ^2^ Although PD‐1/L1 inhibitors have shown incredible therapeutic efficacy and are largely well tolerated, they do not maintain a lasting response in all treated patients. In fact, it is reported that the response rates vary widely. A reliable biomarker will be helpful in predicting the therapeutic efficacy of PD‐1/L1 inhibitors^3^ and selecting potential patients benefiting from PD‐1/L1 inhibitor treatment.

To date, tumor marks, such as tumor mutational burden (TMB),^4^ tumor infiltrating lymphocytes (TILs),^5^ and the presence of microsatellite instability (MSI)^6^ have been the most widely studied predictive biomarkers in the time of PD‐1/L1 inhibitor treatment.^7^ Although PD‐L1 expression is especially important in predicting PD‐1/L1 inhibitor treatment sensitivity, the PD‐L1 expression status was not always clear in cases when limited specimens were obtained before treatment, and it is not suitable for repeated biopsy to avoid the risk of pleural dissemination. However, a lack of consensus on the critical cutoff values of TMB, TILs, and MSI, and the complexity in testing them have limited their clinical applications. In addition to assessing PD‐L1, TMB, TILs, and MSI, simple biomarkers from blood tests evaluating the host immune or nutritional status are also valuable. Among them, neutrophil‐to‐lymphocyte ratio (NLR),^8^ platelet‐to‐lymphocyte ratio (PLR),^9^ and prognostic nutritional index (PNI)^10^ are readily available and inexpensive candidates. The prognostic value of these markers has been widely studied in different kinds of cancers.

PNI is an easily available index based on nutritional and immunological parameters, and has attracted much attention in recent years. PNI was calculated as: 10 × albumin (g/dl) + 0.005 × total lymphocyte count (per mm^3^).^11^ Since the introduction of PNI, many investigators have provided evidence that low PNI predicts cancer prognosis whether patients receive surgery or chemoradiotherapy.^10^, ^12^ The prognostic value of PNI in advanced or metastatic cancer receiving PD‐1/L1 inhibitors has also been explored recently. Some have shown that PNI was associated with prognosis in advanced cancer receiving PD‐1/L1 inhibitors, while others did not. The prognosis of PNI in cancer receiving PD‐1/L1 inhibitors needs further investigation to provide more evidence for simply obtained tumor markers.

Therefore, our study aims to explore the prognostic value of PNI in advanced malignant patients treated with PD‐1/L1 inhibitors.

## 2 MATERIALS AND METHODS

### 2.1 Study selection strategy

Two investigators independently searched PubMed, Embase(via OvidSP), and Web of Science for relevant articles that investigated the prognosis of PNI in malignant patients treated with PD‐1/L1 inhibitors published before October 6, 2021. The searching strategy was: ((prognostic nutritional index) OR (PNI)) AND (((((immunotherapy or immune checkpoint inhibitor) OR (programmed death ligand‐1 inhibitor)) OR (PD‐L1 inhibitor)) OR (programmed death‐1 inhibitor)) OR (PD‐1 inhibitor)). References in eligible studies were also carefully browsed to find potential researches.

Candidates meet the following criteria were regarded eligible: (1) long‐term survival was provided: overall survival (OS) or progression‐free survival (PFS); reported treatment response data including objective response rate (ORR) or disease control rate (DCR) or provided sufficient data to calculate them; and (2) literature published with full text in English. (3) Hazard ratios (HRs) or relative risks (RRs) with 95% CIs were either reported directly or could be extracted from original articles.

Exclusion criteria: (1) studies with insufficient data from which HRs or RRs and 95% CIs could be obtained such as reviews, case or series reports, conference abstracts, or comments; (2) studies in which patients received combined therapy (chemotherapy or target therapy or antiangiogenic therapy); and (3) overlapping or duplicated data.

### 2.2 Data extraction

The following information was extracted and recorded by two investigators independently: first author, country, publication year, patient numbers, cancer type, pretreatment PNI cutoff value, survival analysis mode, and number of patients respond to PD‐1/L1 inhibitors (ORR and DCR). We would use the reported HR with 95% CI if available. Otherwise, data were extracted from the Kaplan–Meier survival curves.^13^ We preferred multivariate analysis results when available, otherwise the univariate analysis results were selected.

### 2.3 Quality assessment

The Newcastle‐Ottawa Scale (NOS) tool was used to evaluate study quality. We considered studies with scores higher than six as high‐quality studies.

### 2.4 Statistical analysis

Prognostic outcomes, including OS and PFS were primary endpoints of this study. HR with 95% CI was pooled to estimate the prognostic value of PNI in advanced cancer patients. RR with 95% CI was combined to explore the risk of PNI in PD‐1/L1 inhibitor treatment ORR and DCR. A fixed‐effects model was adopted when no significant heterogeneity was observed (I^2^ < 50%), which indicated low (*I* ^2^ < 25%) or moderate (*I* ^2^ = 25%–50%) heterogeneity, otherwise a random‐effects model was adopted. Sensitivity analysis was performed to explore the influence of each individual study on OS and PFS. Publication bias was evaluated by funnel plots, Egger's test,^14^ and Begg's test.^15^ We used stata 14.0 software (Stata Corporation) to conduct statistical analyses.

## 3 RESULTS

### 3.1 Characteristics of eligible studies

**FIGURE 1:**
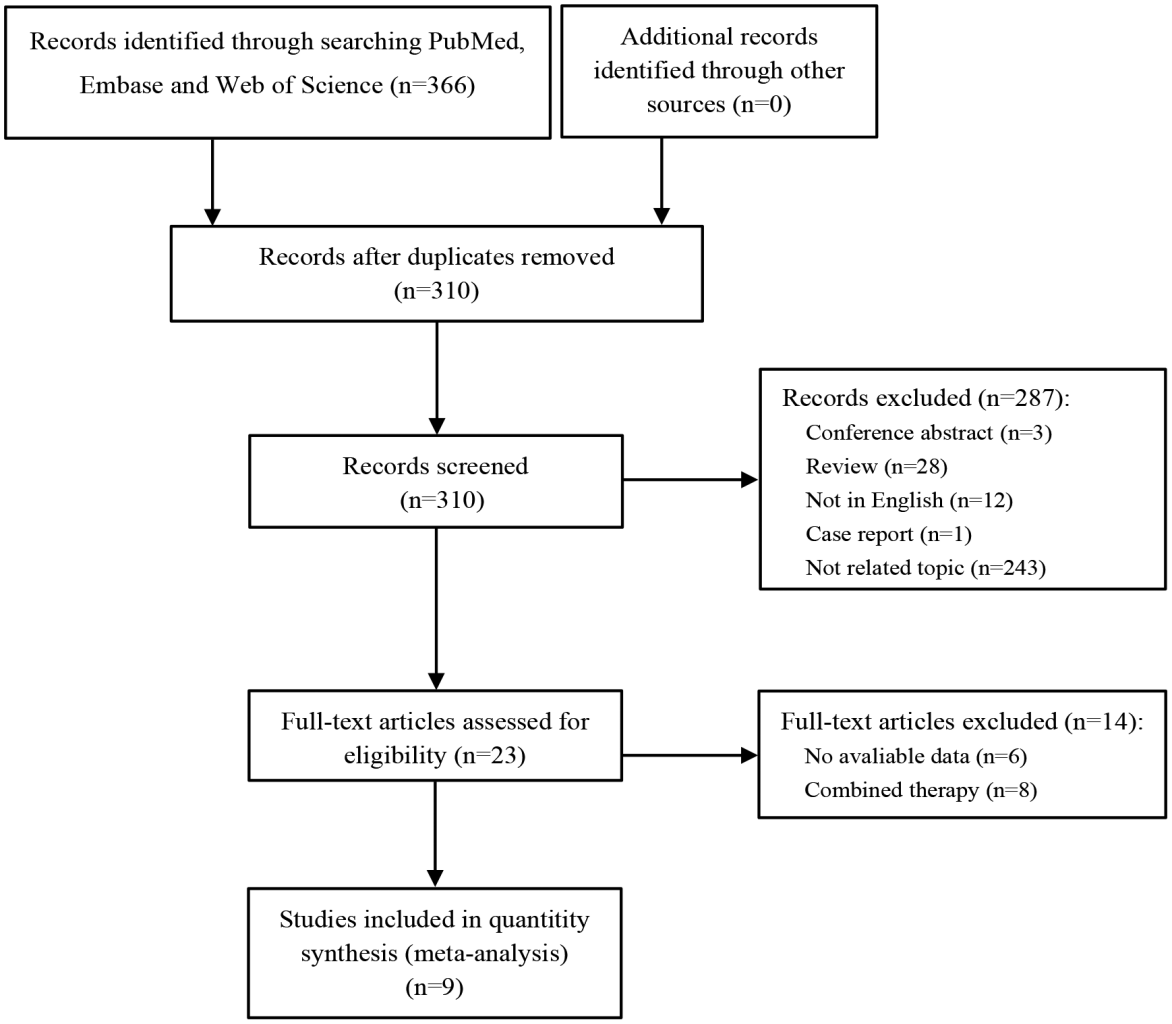
Flow diagram of literature review

A total of 366 records were initially identified by searching three databases. Fifty‐six duplicates were first excluded. After excluding 287 studies by browsing the titles and abstracts, we read the full texts of remaining 23 studies. After excluding six studies with no available data and eight studies in which patients received combined therapy, nine eligible retrospective observational studies^16^, ^17^, ^18^, ^19^, ^20^, ^21^, ^22^, ^23^, ^24^ with available data were finally selected as eligible for this meta‐analysis (Figure 1). HRs with 95% CIs regarding PNI on OS could be obtained from all nine studies directly and data regarding PNI on PFS could be obtained from eight studies.^16^, ^17^, ^19^, ^20^, ^21^, ^22^, ^23^, ^24^ The ORR with 95% CIs could be calculated from three studies^17^, ^18^, ^21^ and the DCR with 95% CIs could also be calculated from two studies.^18^, ^23^ All of nine studies were regarded to have high or moderate quality (NOS: 6–8), the characteristics of which are depicted in Table 1. These studies were published since 2019, with a majority of them being conducted in Japan. The sample size ranged from 24 to 150, among which three were focused on lung cancer, two on urothelial carcinoma, one on esophageal cancer, one on gastric cancer, one on gastric cancer or gastroesophageal junction cancer, and the last one on different kinds of cancer including: melanomas, renal cell carcinoma, NSCLC, Hodgkin lymphoma, head and neck cancer, and transitional cell carcinoma.

**TABLE 1:** Basic characteristics of included studies

First author	Year	Country	Study period	Sample size	Gender (M /F)	Cancer type	PD‐1/L1 inhibitors	Lines of PD‐1/L1 inhibitors	PNI cutoff value	Types of survival outcomes	Nos
Guven et al.	2021	Turkey	Sep 2014 –Jun 2019	150	104/46	Advanced cancer including: melanomas, renal cell carcinoma, NSCLC, Hodgkin lymphoma, head and neck cancer, transitional cell carcinoma	Nivolumab, Atezolizumab, Ipilimumab, Pembrolizumab	1st line 19, 2nd line 58, 3rd line 31, ≥4th line 42	45	OS, PFS	8
Ishiyama et al.	2021	Japan	Jan 2018 –Jun 2020	65	44/21	Metastatic urothelial carcinoma	Pembrolizumab	2nd line 50, ≥3rd line 15	40	OS, PFS	8
Kim et al.	2021	Korea	2016–2019	60	56/4	Advanced ESCC	Nivolumab, Pembrolizumab	2nd line 21, ≥3rd line 39	35.93	OS, PFS	8
Matsubara et al.	2020	Japan	Jan 2018 –Mar 2019	24	17/7	Unresectable advanced NSCLC	Atezolizumab	2nd‐3rd 12, ≥4th 12	48	OS	7
Namikawa et al.	2020	Japan	Oct 2017 –Dec 2019	29	19/10	Unresectable advanced or recurrent gastric cancer	Nivolumab	1st line 29	31.1	OS, PFS	7
Peng et al	2020	China	Jan 2017 –May 2019	102	87/15	NSCLC (IIIB/IV)	Nivolumab, Pembrolizumab, Toripalimab, Sintilimab	1st line 19, 2nd line 51, ≥3rd line 32	45	OS, PFS	6
Takuto et al.	2020	Japan	Dec 2017 –Aug 2019	27	23/4	Metastatic urothelial carcinoma	Pembrolizumab	≥2nd line 27	45	OS, PFS	6
Watanabe et al.	2021	Japan	Oct 2015 –Dec 2019	110	79/31	Advanced or recurrent gastric cancer or gastro‐esophageal junction cancer	Nivolumab	3rd line 70, 4th line 26, ≥4th line 14	40	OS, PFS	8
Zaitsu et al.	2021	Japan	Nov 2016 –Apr 2020	73	52/21	Inoperable lung cancer	Nivolumab, Pembrolizumab, Atezolizumab	1st line 73	43	OS	6

Abbreviations: F, female; M, male; PNI, prognostic nutritional index; OS, overall survival; PFS, progression‐free survival; NSCLC, non‐small cell lung cancer; NOS, Newcastle–Ottawa Scale.

### 3.2 Prognostic impact of PNI on OS

**FIGURE 2:**
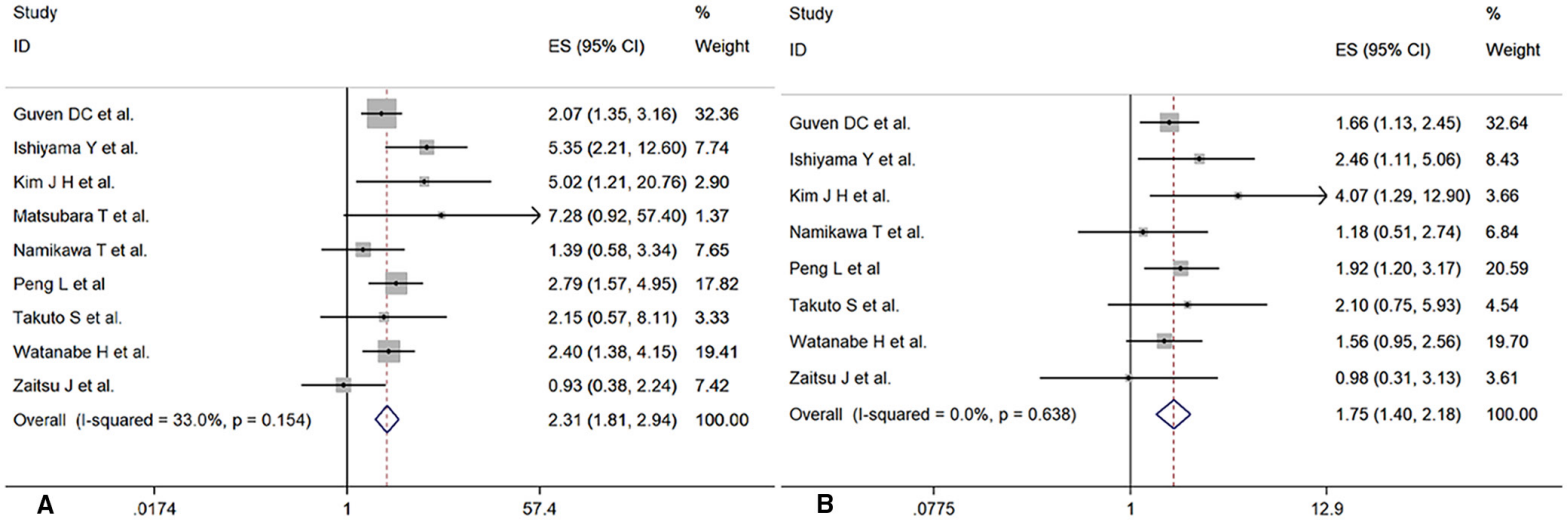
Forest plot for the association between PNI and (A) overall survival (OS), (B) progression‐free survival (PFS)

All nine studies^16^, ^17^, ^18^, ^19^, ^20^, ^21^, ^22^, ^23^, ^24^ consisting of 640 patients were enrolled to analyze the association between PNI and OS. The pooled results suggested a worse OS in patients with lower PNI (HR = 2.31, 95% CI 1.81–2.94, *p* = 0.000), with moderate heterogeneity (*I* ^2^ = 33.0%, *p* = 0.154). (Figure 2A).

### 3.3 Prognostic impact of PNI on PFS

Eight studies^16^, ^17^, ^19^, ^20^, ^21^, ^22^, ^23^, ^24^ consisting of 616 patients were included to analyze the association between PNI and PFS. The pooled results showed that patients with low PNI had shorter PFS (HR = 1.75, 95% CI 1.40–2.18, *p* = 0.000), with no significant heterogeneity (*I* ^2^ = 0%, *p* = 0.638) (Figure 2B).

### 3.4 Sensitivity analysis and publication bias

**FIGURE 3:**
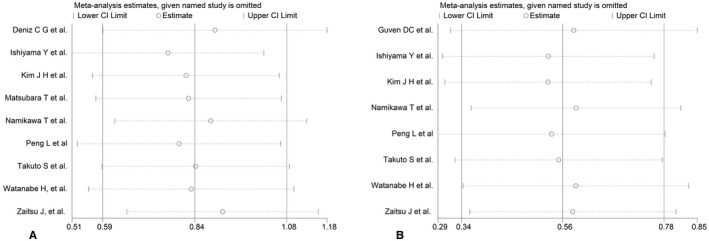
Sensitivity analysis for the association between PNI and (A) overall survival, (OS) and (B) progression‐free survival (PFS)

**FIGURE 4:**
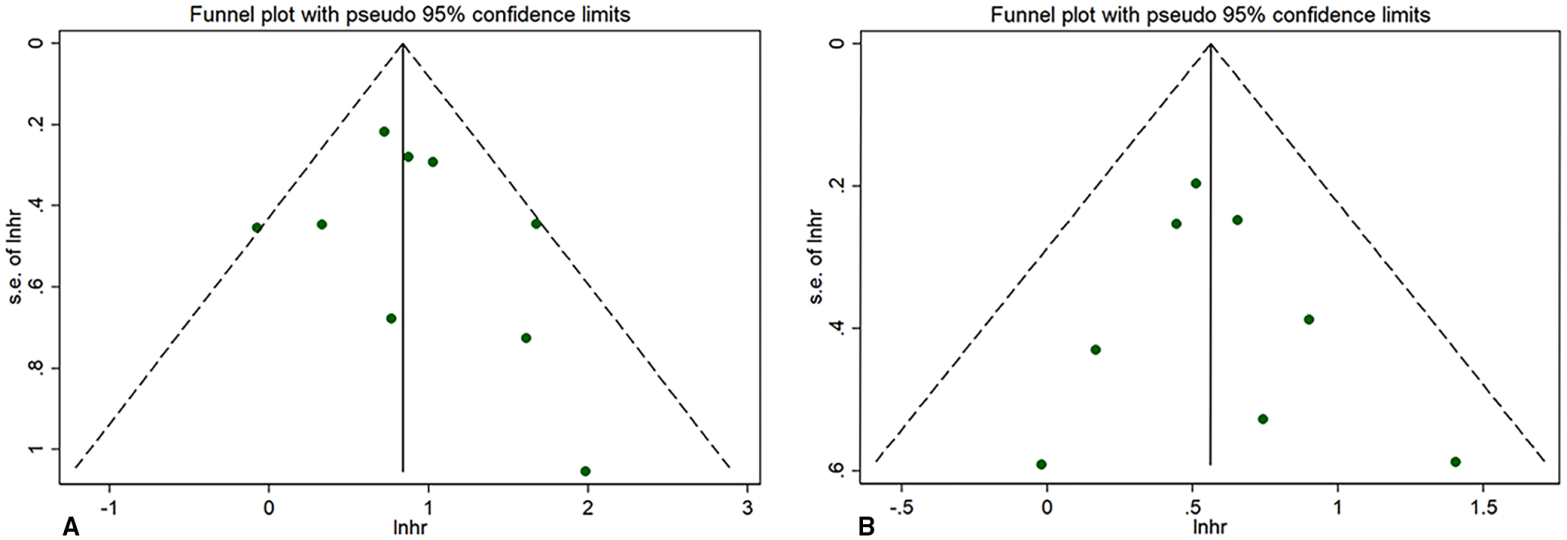
Funnel plot for (A) overall survival (OS) and (B) progression‐free survival (PFS)

**FIGURE 5:**
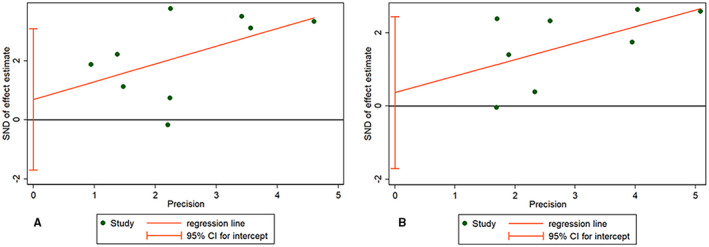
Begg's test for (A) overall survival (OS) and (B) progression‐free survival (PFS)

Sensitivity analysis indicated that each individual study had no significant influence on observed effect size (Figure 3A for OS, Figure 3B for PFS). The funnel plot indicated no apparent asymmetry (Figure 4A,B) and we observed no publication biases in Egger's tests (Figure 5A,B), or Begg's tests (*p* = 0.466 for OS, *p* = 0.902 for PFS). These results indicated no obvious publishing bias.

### 3.5 Subgroup analysis

Subgroup analyses based on related clinical characteristics were conducted to explore the potential source of heterogeneity for OS. Results demonstrated that low‐PNI predicted worse OS in patients regardless of the cut‐off value (≥45 or <45), sample size (≥100 or <100), publication country (Japan or others), or cancer type (NSCLC or others). In the subgroups analysis of Cox regression analysis model, PNI did not predicted OS in univariate group (HR = 1.88, 95% CI 0.95–3.75, *p* = 0.072) (Table 2).

**TABLE 2:** Results of subgroup analysis for impact of PNI on overall survival

Subgroup analysis	Number of studies	Pooled HR( 95% CI)	*p* value	Heterogeneity	
				*I* ^2^ (%)	Ph
Total	9	2.31(1.81, 2.94)	0.000	33.0	0.154
Cut off value					
≥45	4	2.36(1.70, 3.27)	0.000	0	0.600
<45	5	2.25(1.57, 3.23)	0.000	60.2	0.040
Sample size					
≥100	3	2.33(1.74, 3.11)	0.000	0	0.704
<100	6	2.27(1.46, 3.52)	0.000	55.5	0.047
Country					
Japan	6	2.21(1.55, 3.15)	0.000	50.3	0.074
Others	3	2.40(1.72, 3.34)	0.000	0	0.410
Cox regression analysis model					
Univariate	3	1.88(0.95, 3.75)	0.072	6.3	0.344
Multivariate	6	2.38(1.84, 3.08)	0.000	47.0	0.093
Cancer type					
NSCLC	3	2.16(1.35, 3.45)	0.001	64.1	0.062
Other cancers	6	2.37(1.78, 3.14)	0	20.2	0.281

Abbreviations: HR, hazard risk; 95% CI: 95% confidence interval; Ph, *p* value of *Q* test for heterogeneity test; NSCLC, non‐small cell lung cancer; PNI, prognostic nutritional index.

### 3.6 Association between PNI and ORR/DCR

**FIGURE 6:**
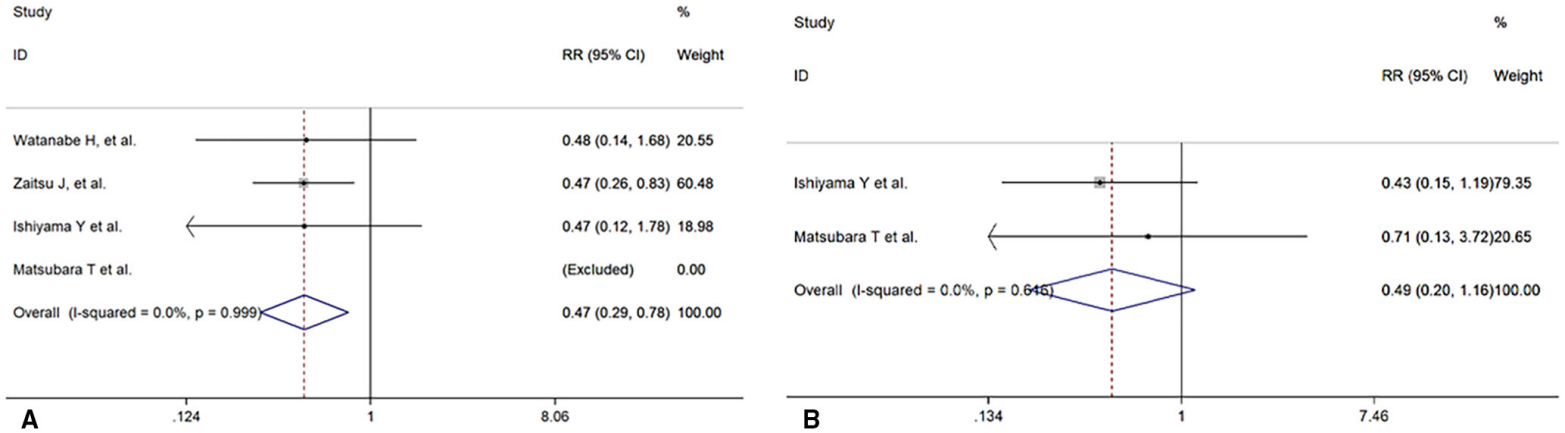
Forest plot for the association between PNI and objective regression rate (ORR) and (B) disease control rate (DCR)

Four studies^17^, ^18^, ^21^, ^24^ explored the influence of PNI and ORR, and a significant relationship between PNI and ORR was observed (RR = 0.47, 95% CI: 0.29–0.78, *p* = 0.003; *I* ^2^ = 0%)(Figure 6A). Similarly, two studies^18^, ^21^ explored the influence of PNI and DCR, but no significant relationship between PNI and DCR was observed (RR = 0.49, 95% CI: 0.20–1.16 *p* = 0.103; *I* ^2^ = 0%)(Figure 6B).

## 4 DISCUSSION

Calculated based on serum albumin and total lymphocyte count, PNI is an easily obtained variable and reflects not only the nutritional status, but also the immune status. Since reported in the 1980s,^11^ PNI has been proven significantly associated with the long‐term survival and treatment responses in various cancers. Currently, studies have explored the prognostic value of PNI in cancers receiving PD‐1/L1 inhibitors. However, no consensus has been reached regarding the prognostic role of PNI in this subject thus far; as far as we knowledge, our meta‐analysis is the first to assess the prognostic value of PNI in advanced cancer patients receiving PD‐1/L1 inhibitors.

We included nine studies with 660 advanced or inoperable cancer patients received PD‐1/L1 inhibitors treatment. To reduce heterogeneity, we excluded studies in which patients received combined therapy and included studies in which patients received PD‐1/L1 inhibitors. All nine studies reported OS data directly regarding the prognostic value of PNI in advanced or inoperable cancer. Among them, five revealed a significant association between low PNI and OS,^17^, ^19^, ^20^, ^21^, ^22^ while the other four did not reach statistical significance. Eight studies^16^, ^17^, ^19^, ^20^, ^21^, ^22^, ^23^, ^24^ reported PFS data directly. Pooled results revealed that a low PNI acted as a negative prognostic factor for both OS (HR = 2.32) and PFS (HR = 1.75). Before us, PNI was shown to act as a significant prognostic factor in different kinds of cancer receiving chemotherapy, surgery, and radiotherapy.^25^, ^26^, ^27^, ^28^ While no studies have comprehensively summarized the prognostic value of PNI in patients receiving PD‐1/L1 inhibitors, our meta‐analysis has substantially increased the evidence on this topic. After combining four articles that explored the influence of PNI and ORR and two that explored the association between PNI and DCR, meta‐analysis revealed that a low PNI was associated with a low ORR (RR = 0.47, *p* = 0.003) but no association with DCR (RR = 0.49, *p* = 0.103). These opposing results between treatment responses could be explained by the limited study numbers, and we believe that the results will change with more studies reported in the future. Another study by Kim et al.^22^ reported no relationship between baseline PNI and DCR after multivariate logistic analysis with an OR = 0.17 (95% CI: 0.03–1.11). We did not include this study in the DCR analysis because no detailed treatment response information between patients with high or low PNI was provided in the original article. Accordingly, low PNI was significantly associated with poor survival outcomes as well as inferior ORR in patients undergoing PD‐1/L1 inhibitors treatment.

PNI reflects both the patients' nutritional and immune status as it is derived from peripheral serum albumin and lymphocyte. Studies have shown that serum albumin and lymphocytes are independent prognostic risk factors for cancer patients. On the one hand, the low PNI is expected to detect malnutrition and is proposed to be pathogenic in the development of cancer‐associated malnutrition, leading to poor performance and increased mortality.^7^ Patients with poor nutritional conditions tend to have a poor immune environment, but low serum albumin does not correlate with protein‐energy malnutrition, and it indicates a high metabolic risk and prediction of cancer morbidity and mortality.^29^ On the other hand, circulating effector cells are the basis for producing natural and therapeutic‐induced anticancer responses. CD8+ T and NK cells directly or indirectly recruit adaptive immune cells by producing chemokines and stimulating antigen‐presenting cells to trigger effective cancer immune monitoring.^30^, ^31^, ^32^ Lymphocytes can also migrate though tissues and infiltrate the tumor microenvironment to change the tumor microenvironment and prevent the occurrence and recurrence of tumors.^33^ Low lymphocyte count may be related to the preexisting immunosuppressive condition, indicating that the host immune response is insufficient. In addition, a low lymphocyte count may be the result of lymphocyte cytokines produced by lymphoma cells, which may be drug‐resistant.^34^

Many factors such as Glasgow Prognostic Score, NLR, PLR, body mass index, and C‐reactive protein have been reported to reflect the immune or nutrition status.^35^ While contradictions often exist when these markers were applied in clinical practices, for that the value of one marker was often evaluated in certain circumstance for the restricted applicability. There still lacks efficient and widely available factors in monitoring the immune‐nutrition conditions. Besides, along with the widely application of PD‐1/L1 in cancer patients, factors in the immune‐nutrition field in predicting treatment response as well as long‐term survival were desperately needed. PNI has shown excellent prospects when comprehensively compared with the other factors in the background of PD‐1/L1 treatment.^36^, ^37^

Some limitations should not be ignored in interpreting our results. First, the retrospective observational property of all the included articles and limited sample size led to the decline in the level of evidence. Second, not all of the survival analyses were conducted by multivariate analysis; thus, some confounding factors may exist. Third, heterogeneity was another issue that should be addressed because most studies were from Asian countries, and ethnic bias may exist. Furthermore, the PNI cutoff values varied between 31.1 and 48 in our meta‐analysis, which may significantly increase clinical heterogeneity. Last but not least, there were significant differences in cancer types, types and lines of PD‐1/L1 inhibitors in different studies, which may lead to significant clinical heterogeneity. Due to insufficient data available in the original article, we were unable to further conduct subgroup analysis based on PD‐1/L1 inhibitor subtypes and cancer types.

## 5 CONCLUSION

In conclusion, low PNI was significantly associated with reduced long‐term survival and treatment responses in patients with advanced cancer treated with PD‐1/L1 inhibitors. Therefore, PNI can be used as an auxiliary tool to predict prognosis and identify high‐risk patient receiving PD‐1/L1 inhibitors. More well‐designed and large sample studies are urgently needed to verify our results.

## CONFLICT OF INTEREST

The authors declare no conflict of interest.

## AUTHOR CONTRIBUTIONS

Qinghua Zhou and Pengfei Li conceived and designed the study; Pengfei Li, and Long Tian collected and analyzed the data; Pengfei Li and Yutian Lai contributed the materials/analysis tools and wrote the manuscript. All authors reviewed and approved the manuscript prior to submission.

## ETHICS STATEMENT

No ethical approval was required for the meta‐analysis as all data originated from previously published studies.

## Data Availability

Data sharing is not applicable to this article as no new data were created or analyzed in this study.
